# Hypertension-Focused Medication Therapy Management: A Collaborative Pilot Program Uniting Pharmacists, Public Health, and Health Insurers in Wisconsin

**DOI:** 10.5888/pcd17.200058

**Published:** 2020-09-10

**Authors:** Hailey Thompson, Lena Swander, Rebecca Cohen, Alan Lukazewski, Tim Bartholow, Mary Pesik, Kari Trapskin

**Affiliations:** 1 Pharmacy Society of Wisconsin, Madison, Wisconsin; 2 Wisconsin Department of Health Services, Division of Public Health, Madison, Wisconsin; 3 NeuGen, Madison, Wisconsin

## Abstract

Heart disease and stroke are leading causes of death and disability in the United States, and high blood pressure is a major risk factor for both. Community pharmacists are readily positioned to improve cardiovascular health through services such as medication therapy management and self-management education. In 2018, the Pharmacy Society of Wisconsin, the Wisconsin Division of Public Health, and NeuGen, a not-for-profit health insurer, piloted a pharmacist-led medication therapy management program for people with hypertension in partnership with 8 community pharmacies. We evaluated changes in use of blood pressure self-management tools and barriers to antihypertensive medication adherence before and after medication therapy management services. Participant satisfaction was also assessed for the 59 participants at the end of the program. We observed improvements in self-reported use of self-management tools, reductions in medication adherence barriers, and high satisfaction with pharmacist care. This collaborative pilot resulted in sustainable reimbursement for participating pharmacies delivering medication therapy management services to eligible NeuGen members.

**Summary**

**What is already known on this topic?**

Pharmacy-delivered medication therapy management can improve health outcomes. However, evidence across studies varies because of the inconsistency in operationalization of service delivery and population heterogeneity.

**What is added by this report?**

We evaluated a collaborative medication therapy management pilot program for people with hypertension in Wisconsin. We demonstrated improvements in self-reported use of blood pressure self-management tools and barriers to medication adherence.

**What are the implications for public health practice?**

Sustainable reimbursement mechanisms were established for select pharmacies delivering medication therapy management to members of a private health plan. Other public health entities might consider replicating our collaborative pilot model to secure reimbursement for pharmacist-delivered services.

## Introduction

Heart disease and stroke are leading causes of death and disability in the United States, and high blood pressure is a major risk factor for both (1). The Centers for Disease Control and Prevention recommends pharmacist-delivered medication therapy management (MTM) services to improve cardiovascular health for those with hypertension (2). MTM is an umbrella term for medication services that include, but are not limited to, comprehensive medication review/assessment (CMR/A), the creation of medication-related action plans, pharmacist referral or intervention, and documentation and follow-up (3). Evidence suggests that pharmacist-led interventions with elements of MTM delivered in a community pharmacy setting are effective in helping patients with hypertension lower their blood pressure and even achieve control. A 2014 systematic review and meta-analysis of randomized controlled trials associated community pharmacist-led interventions with significant reductions in systolic and diastolic blood pressure compared with usual care (4). Interventions included pharmacological components (eg, identifying adverse drug effects and prescribing issues), nonpharmacological components (eg, providing education on healthy lifestyle changes), or both. Early evidence also suggests that when pharmacists are engaged in education about self-measured blood pressure monitoring, patients with hypertension achieve better blood pressure outcomes (5–7).

In 2017, the Association of State and Territorial Health Officials (ASTHO) announced a year-long learning collaborative with the state public health agency focused on improving population-level blood pressure control. (8) The ASTHO learning collaborative required that state public health agencies partner with private health insurers to improve cardiovascular outcomes in an innovative manner. The collaborative’s design allowed Wisconsin’s grant recipients to contribute to evidence surrounding pharmacist-delivered MTM and its impact on cardiovascular health.

## Purpose and Objectives

From December 2017 through September 2018, the Wisconsin Department of Health Services’ Division of Public Health partnered with the Pharmacy Society of Wisconsin and NeuGen (https://www.neugenhealth.com/), a not-for-profit health insurer, to implement and evaluate a pharmacist-led MTM pilot program for people with hypertension as part of the ASTHO learning collaborative. The pilot program’s design was informed by the Pharmacists’ Patient Care Process (PPCP) model and by evidence supporting pharmacist-led interventions in community pharmacy settings. PPCP is a framework created by the Joint Commission of Pharmacy Practitioners to guide pharmacist collaborative and patient-centered care to improve health and medication outcomes (9). In addition to a CMR/A, our pilot also included pharmacist-led education about self-measured blood pressure. Our evaluation assessed changes resulting from pharmacist-delivered MTM services in participant knowledge and health beliefs about hypertension, use of blood pressure self-management tools (logs and monitors), and medication adherence barriers.

**Partnerships and established MTM program infrastructure**. The Pharmacy Society of Wisconsin supports more than 4,000 pharmacists, technicians, and pharmacy students in Wisconsin. In 2008, the society launched the Wisconsin Pharmacy Quality Collaborative (WPQC) to align incentives for pharmacies and health insurers. WPQC currently comprises 187 pharmacies and 340 pharmacists accredited and certified by the Society. WPQC pharmacists complete training and receive certification to resolve drug therapy problems, improve adherence, and engage people in their care through MTM service delivery. All WPQC-accredited pharmacies have a private area for MTM service delivery.

The Wisconsin Department of Health Services Forward Health program (Medicaid) covers MTM in the form of CMR/A services when provided to eligible members by WPQC pharmacists. In this context, CMR/A involves a WPQC pharmacist evaluating a patient’s health status and medications to identify and resolve medication-related issues. If the pharmacist and patient identify concerns, they work with the primary care provider to resolve them. Partnerships with the Wisconsin Department of Health Services and with federal and private grants over the last decade have aided the promotion and expansion of the WPQC program and MTM service delivery.

NeuGen insures more than 105,000 people in Wisconsin. NeuGen and the Pharmacy Society of Wisconsin connected their efforts through mutual relationships with the Wisconsin Department of Health Services’ Division of Public Health. The pilot program described and evaluated here benefitted from existing relationships and MTM program infrastructure to improve chronic disease outcomes.

## Intervention Approach

**Pharmacy selection and participant eligibility**. NeuGen facilitated pharmacy selection for the pilot program by identifying clusters of members with hypertension who filled prescriptions at WPQC pharmacies. We defined member eligibility as any adult (aged ≥18 y) NeuGen health plan member with diagnosed hypertension who filled an antihypertensive medication prescription at a WPQC pharmacy during the 12 months before April 2018. By using claims data, NeuGen identified eligible members and associated prescription fills with corresponding WPQC pharmacies. Partners ranked WPQC pharmacies by the total associated, eligible member count. The Pharmacy Society of Wisconsin used this list to recruit 8 WPQC pharmacies: 4 pharmacies in Kenosha County, 3 in Sauk County, and 1 in Langlade County. Kenosha is an urban county in the southeast corner of Wisconsin. Sauk and Langlade are rural counties in the south-central and northern parts of the state, respectively.

Participating pharmacies represented 145 NeuGen health plan members. Pharmacists and staff at these locations participated in an orientation webinar that introduced program design and implementation. Pharmacists were also asked to view e-learning modules on self-measurement of blood pressure and accurate blood pressure management (10,11). The Pharmacy Society of Wisconsin provided hypertension-specific clinical toolkits and adherence training and tools to work with patients to identify solutions to self-reported adherence barriers. NeuGen incentivized participating pharmacies by providing reimbursement for MTM service delivery, a tablet computer to facilitate survey completion, and a stipend for marketing and data collection.

**Recruitment**. NeuGen contacted 145 eligible members via mail, informing them of the program, that their pharmacy was participating, and offering them a $25 gift card incentive to a local gas station/convenience store for completion of 2 in-person visits with the pharmacist. Interested members were asked to contact their pharmacy to learn more. Pharmacy staff members made follow-up telephone calls to nonrespondents 2 to 4 weeks after the mailing. Of 145 eligible NeuGen members, 42% (N = 61) agreed to participate.

**MTM service delivery and participant survey**. Pharmacists successfully delivered 2 in-person MTM visits to 59 participants from May through September 2018. The time between participant visits varied from approximately 4 to 6 weeks. At the beginning of each visit, pharmacists administered a participant survey that documented self-reported barriers to antihypertensive medication adherence and use of blood pressure self-measurement/monitoring tools. The survey was followed by the completion of a CMR/A service. Throughout the service, pharmacists provided verbal education about healthy lifestyle changes, educated participants on blood pressure self-measurement and monitoring, and used motivational interviewing techniques to address adherence barriers. Classifying adherence barriers into 5 domains (system, understanding, motivation, recall, and financial) aided pharmacists in generating adherence solutions. Pharmacists also provided the following tools to patients: personal medication list, medication action plan, self-measurement of blood pressure education form, and a log for recording home blood pressure readings. Following both visits, participants completed an online exit survey on satisfaction. To ensure HIPAA (Health Insurance Portability and Accountability Act) compliance, pharmacists submitted anonymous survey data directly to NeuGen via SurveyMonkey (www.surveymonkey.com/). If needed, pharmacists communicated with participants’ primary care providers to optimize medication therapy following both visits. Pharmacists gave each participant a blood pressure monitor paid for by NeuGen and the Department of Health Services.

## Evaluation Methods

We conducted McNemar tests of correlated proportions on paired survey data collected from May through September 2018 to evaluate changes in participant-reported use of self-management tools. We also quantified changes in proportions of participants who reported experiencing barriers to antihypertensive medication adherence before and after MTM service delivery. Finally, we assessed overall participant satisfaction with pharmacist-provided care. We conducted quantitative analyses in SAS version 9.4 (SAS Institute Inc) and Microsoft Excel version 14.0 (Microsoft Corp). The Pharmacy Society of Wisconsin adapted pharmacist satisfaction questions from Ried et al (12) and medication adherence tools from the Brief Medication Questionnaire (13) (Box). We did not conduct prospective power calculations because of the rapid planning and execution of the pilot required by the ASTHO learning collaborative’s timeline.

**Box. Questions in Pharmacist Satisfaction Survey**

How would you rate the overall care you received from your pharmacists? [Answer options were very poor, poor, fair, good, or very good.]

Do you use any of the following to help you manage your blood pressure? [Answer options were yes or no.]

- A monitor to check my blood pressure at home
- A log to keep track of my blood pressure readings
- A log to track the days I take my blood pressure medication

How much difficulty do you have in the following areas with your blood pressure medication? [Answer options were none, a little, some, or a lot.]

- Remembering my medication dosage(s)
- Remembering if I took my medication
- Paying for my medication
- Refilling my medication
- Unwanted side effects from my medication
- Reading my medication bottles
- Other concerns or problems with my medications

## Results

From May through September 2018, 28 women and 33 men participated in the pilot program. Ages ranged from 35 to 76, with a mean age of 60. Fifty-nine participants completed both visits.

**Self-reported use of self-management tools**. We observed improvements in self-reported use of self-management tools (Table). Following program participation, patients were more likely to report use of a log to track blood pressure readings (χ^2^ [1, N = 59] = 35.1, *P* < .001). Participants were also more likely to report use of a log to track antihypertensive medication use (χ^2^ [1, N = 59] = 8.1, *P* = .045). Participants were also more likely to report use of self-measuring blood pressure monitors (χ^2^ [1, N = 59] = 39.0, *P* < .001).

**Table:** Participant (N = 59) Characteristics Related to Antihypertensive Medication Therapy and Blood Pressure Management Practices, Before and After Implementation of Medication Therapy Management Services, Wisconsin, May–September 2018^a^

Characteristic	Before	After
**Experienced adherence barrier^b^**		
Remembering dosage	10	5
Remembering to take medications	10	4
Reading medication bottles	8	2
Medication side effects	8	2
**Responded yes to use of blood pressure management techniques**		
Monitor blood pressure at home	14	53
Keep a blood pressure reading log	14	45
Keep a blood pressure medication log	3	14

a Values are number of participants.

b Only categories with at least 1 participant reporting are shown. Includes reports of any level of difficulty (Box).

**Adherence barriers**. The number of patients experiencing adherence barriers decreased across all categories after the second visit (Table). Participants most frequently reported remembering dosage and remembering to take medications as barriers to antihypertensive medication adherence. The proportion of participants who reported experiencing any level of difficulty remembering their medication dosage decreased by 50% following MTM service delivery. The proportion of participants who experienced difficulty remembering to take their antihypertensive medications decreased by 60%. Participation in the adherence barrier questions was not powered for statistical comparison of pre/post results.

**Satisfaction and participant engagement in self-management**. Following MTM delivery, 58 participants rated the pharmacist’s overall care and ability as very good. Most (58) also agreed or strongly agreed with the statement that, as a result of their participation in the program, they were going to take a more active role in managing their blood pressure.

## Implications for Public Health

We created, implemented, and evaluated an MTM pilot program in Wisconsin that showed early indications of the positive impact pharmacists can have on blood pressure self-management. We observed reductions in self-reported barriers to adherence to antihypertensive medication therapy and increased use of self-management tools. Moreover, participants reported high satisfaction with their pharmacist’s care overall. NeuGen indicated that member engagement for this collaborative pilot was considerably higher than for pilot interventions they implemented alone. When patient, pharmacist, and payer incentives are aligned, sustainable programs with demonstrable benefits are created. Collaborative programs between pharmacists, public health, and health insurers contextualize and localize existing evidence that MTM services improve cardiovascular-related health outcomes.

Our study had several limitations. The number of pharmacies and participants was modest and limited our ability to conduct more rigorous analyses of clinical outcomes, particularly on blood pressure readings. Additionally, our participants were drawn from a nonrandom cluster sample of NeuGen members and likely shared similar social, educational, economic, and cultural backgrounds. Pharmacist selection was also based on nonrandom clustering of NeuGen members with hypertension, and aided by Pharmacy Society of Wisconsin recruitment. Finally, the short time frame of the ASTHO learning collaborative did not allow exploration of longitudinal outcomes. Despite these limitations, our pilot program galvanized and matured interagency relationships to improve population health in Wisconsin. In 2019, NeuGen extended its collaboration with the Pharmacy Society of Wisconsin and the Wisconsin Department of Health Services Division of Public Health by launching another MTM program for members with comorbid chronic conditions (hypertension, prediabetes, diabetes, and hyperlipidemia). NeuGen continues to reimburse WPQC pharmacists for MTM service delivery and to provide technology support, blood pressure monitors, and gift card incentives to qualifying members.
